# Intestinal microbiota modulates pancreatic carcinogenesis through intratumoral natural killer cells

**DOI:** 10.1080/19490976.2022.2112881

**Published:** 2022-08-18

**Authors:** Qin Yu, Rachel C. Newsome, Mark Beveridge, Maria C. Hernandez, Raad Z. Gharaibeh, Christian Jobin, Ryan M. Thomas

**Affiliations:** a Department of Medicine, University of Florida College of Medicine, Gainesville, Florida, USA; b Department of Surgery, University of Florida College of Medicine, Gainesville, Florida, USA; c Department of Infectious Diseases and Immunology, University of Florida College of Medicine, Gainesville, Florida, USA; d Department of Anatomy and Cell Biology, University of Florida College of Medicine, Gainesville, Florida, USA; e Department of Molecular Genetics and Microbiology, University of Florida College of Medicine, Gainesville, Florida, USA

**Keywords:** Pancreatic cancer, pancreatic ductal adenocarcinoma, microbiome, immune response, natural killer cells

## Abstract

Preclinical data demonstrate that the gut microbiota can promote pancreatic ductal adenocarcinoma (PDAC), but mechanisms remain unclear. We hypothesized that intestinal microbiota alters anti-tumor innate immunity response to facilitate PDAC progression. Human PDAC L3.6pl cells were heterotopically implanted into *Rag1^−/−^* mice after microbiota depletion with antibiotics, while syngeneic murine PDAC Pan02 cells were implanted intrapancreatic into germ-free (GF) C57BL/6 J mice. Natural killer (NK) cells and their IFNγ expression were quantitated by flow cytometry. NK cells were depleted in vivo using anti-Asialo GM1 antibody to confirm the role of NK cells. Bacteria-free supernatant from SPF and GF mice feces was used to test its effect on NK-92MI cell anti-tumor response *in vitro*. SPF and ex-GF mice (reconstituted with SPF microbiota) developed larger PDAC tumors with decreased NK cell tumor infiltration and IFNγ expression versus GF-*Rag1^−/−^*. Microbiota-induced PDAC tumorigenesis was attenuated by antibiotic exposure, a process reversed following NK cell depletion in both *Rag1^−/−^* and C57BL/6 J mice. Compared to GF, SPF-*Rag1^−/−^* abiotic stool culture supernatant inhibited NK-92MI cytotoxicity, migration, and anti-cancer related gene expression. Gut microbiota promotes PDAC tumor progression through modulation of the intratumoral infiltration and activity of NK cells.

## Introduction

Pancreatic ductal adenocarcinoma (PDAC) accounts for more than 85% of pancreatic cancer cases, is the third leading cause of cancer-related death in the United States, and has a dismal 5-year survival rate of <10% for all stages combined.^1,2^ Treatment difficulties likely arise from the highly immunosuppressive tumor microenvironment but the underlying mechanisms responsible for this phenomenon are unclear.^3^ The intestinal microbiota, composed of trillions of microorganisms, has both beneficial and deleterious roles in various human diseases, including pancreatic cancer.^4,5^ A recent study by Kartal et al. reported a fecal microbiota signature that can discriminate between healthy individuals and patients with PDAC, prior to their diagnosis. How this signature corresponds with the process of pancreatic carcinogenesis is an area of active research.^6,7^ Additionally, studies have reported that the gut microbiota can modulate the immunosuppressive intratumoral environment in PDAC through an adaptive immune response.^8,9^ These studies evaluated the adaptive immune system in PDAC with limited focus on the role of the innate immune system. Previously, we reported that the gut microbiota can enhance PDAC progression independent of adaptive immunity, suggesting that innate cells may also be involved in microbiota-dependent PDAC development.^10^

Natural killer (NK) cells, an important group of innate cytotoxic lymphocytes, were first identified for their tumor killing ability^11,12^ and are now known to be essential in host immunity against various malignancies,^13^ including PDAC.^14^ They elicit anti-tumor function through various mechanisms including activation receptor-based killing and cytokine production for immune modulation and recruitment.^15,16^ Once activated, NK cells induce target cell apoptosis through lytic granule-mediated pathways. Moreover, NK cells secrete numerous cytokines and chemokines, such as interferon gamma (IFNγ) and tumor necrosis factor alpha (TNFα),^16^ which trigger the activation and recruitment of other innate and adaptive immune cells. Prior studies have demonstrated that NK cell tumor infiltration and their subsequent activation positively correlate with PDAC patient survival.^17,18^ In advanced stages of PDAC, however, the anti-tumor effect of NK cells appears to be lost, as evidenced by decreased IFNγ expression, which is critical for NK cell activation and adaptive immune cell recruitment.^17,19,20^

While it is well documented that the intestinal microbiota interacts with tissue-resident immune cells,^21^ a microbial role for systemic immune modulation likely relies on signaling independent of a specific organ.^22^ Such crosstalk may be related to microbial-derived metabolites, cellular byproducts, or small molecules.^23^ These microbial components have the capacity to enter the host systemic circulation to modulate immune response thereby influencing cancer development.^24^ However, sparse information about the interplay between intestinal microbiota and NK cells in cancer is available. Herein, we report that tumor-infiltrating NK cells are important for the crosstalk between gut microbiota and PDAC progression. Bacterial-derived culture supernatant can modulate NK cell anti-tumor immunity. These data support a connection between intestinal microbiota and PDAC development through modulation of NK cell anti-tumor activity via microbial-derived components.

## Results

### Gut microbiota-enhanced pancreatic cancer progression associates with decreased intratumoral NK cell infiltration and activation

We previously reported that the gut microbiota promotes PDAC development independent of host adaptive immunity in a remote (i.e. independent from a tumor or organ-resident microbiome) manner.^10^ To determine which innate immune population may be involved in the remote gut microbiota-PDAC modulation, we first utilized our previously published RNAseq dataset.^10^ Utilizing the TIMER2.0 platform-based^25–27^ immune estimation through CIBERSORT algorithm, there was predicted decreased intratumoral NK cells in PDAC xenografts in microbiota-intact mice compared to mice with antibiotic-mediated depletion (Abx) of the gut microbiota (0 and 7.9% for control and Abx-treated NOD-SCID mice, respectively; Fig. S1A). In addition, a query of The Cancer Genome Atlas (TCGA), demonstrates longer overall survival in patients with high intratumoral NK cell infiltration compared to low (Fig. S1B, *p* = .02). Given emerging evidence of NK cells in cancer treatment^28,29^ and this unexplored area in the PDAC-microbiome literature, we pursued this line of investigation for our work. We therefore hypothesized that the gut microbiota inhibits tumor infiltrating NK cells and therefore promotes PDAC progression.

**Figure 1.**
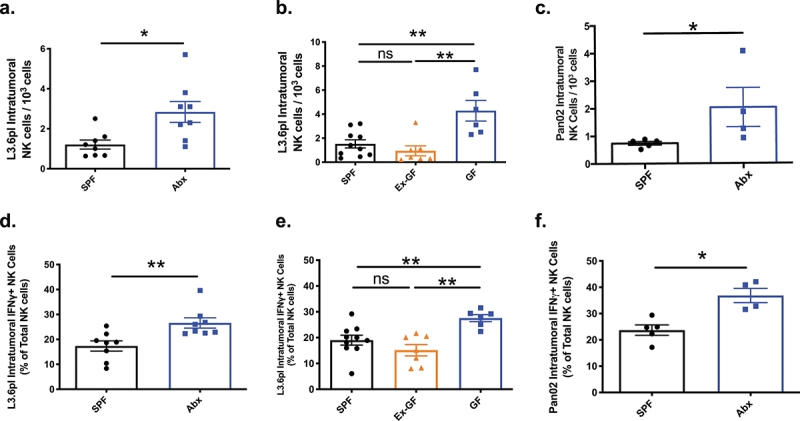
Gut microbiota mediates intratumoral NK cell infiltration and activity in immunocompromised and immunocompetent mice bearing pancreatic cancer xenografts. Heterotopic or orthotopic PDAC xenografts were established as described. At the time of xenograft harvest, tumors were dissociated into single cell suspensions and total NK cell infiltration relative to 10^4^ total tumor cells dissociated were analyzed by flow cytometry. The *Rag1^−/−^* mice bearing human L3.6pl PDAC xenografts had decreased NK cell infiltration in specific pathogen-free (SPF) mice compared to those with microbiota depletion with antibiotics (Abx, A). Likewise, SPF-*Rag1^−/−^* mice had decreased intratumoral NK cell infiltration compared to germ-free (GF) *Rag1^−/−^* mice and the increased intratumoral NK cell infiltration seen in the gnotobiotic mice was reversed with reconstitution of the microbiota of GF-*Rag1^−/−^* mice with stool derived from SPF-*Rag1^−/−^* mice (“Ex-GF”, B). Finally, the presence of a microbiota in immunocompetent C57BL/6 J mice bearing orthotopic syngeneic Pan02 PDAC xenografts also resulted in decreased intratumoral NK cell infiltration compared to Abx-mediated microbiota depletion in C57BL/6 J mice (c). To determine if differences in NK cell infiltration into PDAC tumors also correlated with differences in activation as measured by interferon gamma (IFNγ) expression, flow cytometry for IFNγ was performed on the intratumoral NK cell population. This demonstrated that the presence of a microbiota also inhibited the activation of intratumoral NK cells in the SPF-Rag1^−/−^ (d, e), Ex-GF-Rag1^−/−^ (e), and SPF-C57BL/6 J models (f) compared to their Abx treated (d, f) or GF **(E)** cohorts. (*) *p* < .05, (**) *p* < .01.

To test this hypothesis, we first utilized germ-free or Abx-exposed *Rag1^−/−^* mice, and heterotopically established PDAC xenografts in the flank of mice using human L3.6pl cells. 16S PCR of DNA isolated from stool just prior to the heterotopic establishment of PDAC xenografts confirmed microbiota depletion in representative examples of Abx-exposed mice (Fig. S2A) or absence in germ-free (GF) mice (Fig. S2B). At study endpoint of 28 days, tumors were harvested, weighed and measured, dissociated into single-cell suspensions, and flow cytometry performed to quantitate NK cell infiltration. Compared to Abx-*Rag1^−/−^* mice, SPF-*Rag1^−/−^* with an intact microbiota had a 57.4% reduction in intratumoral NK cell infiltration (*p* = .01; Figure 1a). To control for any potential confounding effects of Abx, L3.6pl xenografts were similarly established in GF, Ex-GF (GF mice gavaged with stool from SPF mice; Fig. S2B), and SPF-*Rag1^−/−^*. The presence of a microbiota in either SPF-*Rag1^−/−^* or the Ex-GF mice likewise resulted in a 64.4% (*p* = .005) and 78% (*p* = .008) reduced intratumoral NK cells infiltration compared to GF mice, respectively (Figure 1b). Finally, in order to account for NK cell interactions with the adaptive immune system,^30^ we extended our investigation to immunocompetent C57BL/6 J mice orthotopically implanted with the syngeneic PDAC cell line, Pan02. Ablation of the gut microbiota with antibiotics was accomplished as described above. In agreement with the previous models, presence of a microbiota attenuated intratumoral NK cell infiltration into the PDAC xenografts by 63.7% (*p* = .02; Figure 1c). In each of these models, PDAC xenograft volume and weight were greater in microbiota-intact mice (SPF-*Rag1*^−/−^, Ex-GF-*Rag1*^−/−^, SPF-C57BL/6 J; Fig. S3A-C, respectively) compared to microbiota-depleted (Abx-*Rag1*^−/−^, Abx-C57BL/6 J) or gnotobiotic (GF-*Rag1*^−/−^), confirming the inverse relationship between PDAC xenograft progression and anti-tumor NK cell infiltration. Consistent with Pushalkar et al.,^8^ gut microbiota also decreased adaptive intratumoral immune response in SPF-C57BL/6 J mice but was not statistically significant (Fig. S4A-B). Flow cytometry gating strategy to quantitate immune cell populations is illustrated in Fig. S5.

A primary indicator of NK cell activation is IFNγ production.^31^ To elucidate if reduced intratumoral NK cell infiltration is also associated with decreased NK cell activation, intratumoral NK cell population was isolated and IFNγ expression determined by flow cytometry. In SPF-*Rag1*^−/−^ mice bearing L3.6pl PDAC xenografts, there was a 34.7% reduction in NK cell IFNγ expression compared to microbiota-depleted Abx-*Rag1*^−/−^ mice (*p* = .002; Figure 1d). Intratumoral NK cells of SPF-*Rag1*^−/−^ and Ex-GF-*Rag1*^−/−^ mice had 31% (*p* = .005) and 45% (*p* = .001) reduced IFNγ-expressing NK cells compared to GF-*Rag1*^−/−^ mice, confirming the inhibitory capability of intestinal bacteria (Figure 1e). Finally, immunocompetent C57BL/6 J mice likewise demonstrated 35.7% (*p* = .016) decreased intratumoral NK cell activation by IFNγ(+) expression in the presence of an intestinal microbiota (SPF-C57BL/6 J) compared to microbiota-depleted Abx-C57BL/6 J mice (figure 1f). These findings indicate that gut microbiota-mediated pancreatic cancer progression is associated with decreased intratumoral infiltration and activation of NK cells regardless of the presence of adaptive immunity.

### Anti-PDAC effect of gut microbiota depletion is dependent on NK cells

**Figure 2.**
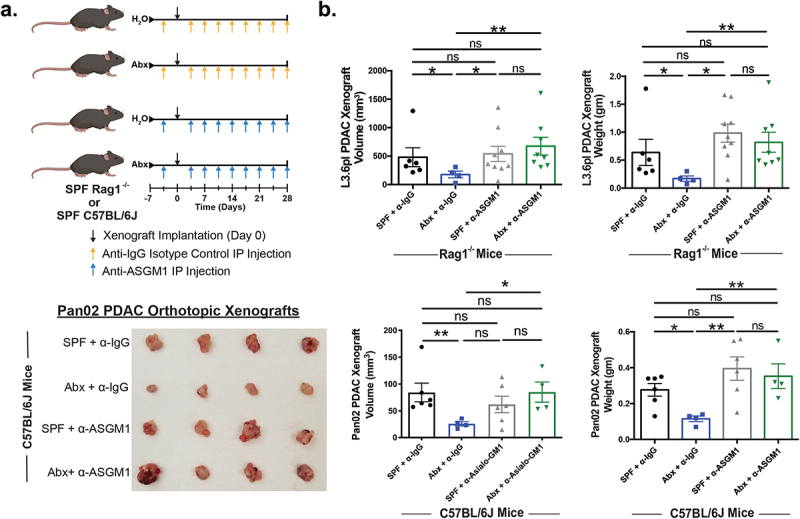
Anti-PDAC effect of gut microbiota depletion is dependent on NK cells. SPF-*Rag1^−/−^* (n = 10) or C57BL/6 J (n = 7) mice were given water or antibiotic cocktail (Abx) to deplete their gut microbiota (a). NK cells were depleted with twice weekly intraperitoneal (IP) injection of anti-ASGM1 antibody or anti-IgG isotype control antibody starting three days prior to PDAC xenograft implantation (*Rag1^−/−^*: subcutaneous L3.6pl; C57BL/6 J: intrapancreatic Pan02). At endpoint, NK cell depletion abrogated the anti-tumor effect of microbiota depletion in both *Rag1^−/−^* (b) and C57BL/6 J mice, representative Pan02 xenografts are shown (c). There was one premature death in the L3.6pl SPF + anti-IgG cohort and differences in cohort size for the remaining cohorts was due to failure of xenograft engraftment with no evaluable tumor to measure and were thus excluded from analysis. (*) *p* < .05, (**) *p* < .01.

To establish the role of NK cells in microbiota-mediated PDAC progression in both immunodeficient and immunocompetent mouse models, we depleted these cells using an anti-ASGM1 antibody (Figure 2a). Flow cytometry analysis confirmed highly efficient depletion of the NK cell population (CD45+/CD3-/NK1.1+) in the anti-ASGM1 cohort compared to the anti-IgG isotype control cohort in both the spleen (0.97% vs. 35.1%, respectively) and L3.6pl xenograft (0.13% vs. 29.1%, respectively; Fig. S6A-B). Interestingly, the anti-PDAC effect of microbiota depletion was abolished following antibody-mediated NK cell depletion in *Rag1*^−/−^ mice bearing L3.6pl xenografts (Figure 2b). Finally, to extend our observations to immunocompetent mice, the NK cell population was again depleted utilizing anti-ASGM1 antibody in C57BL/6 J mice bearing orthotopic syngeneic Pan02 tumors. NK cell depletion in this model also eliminated the anti-tumor phenotype of gut microbiota depletion (Figure 2c). These findings suggest that the anti-PDAC effect of gut microbiota depletion is dependent on NK cells in both PDAC models.

### Microbial-derived components alter NK cell anti-PDAC activity in vitro

**Figure 3.**
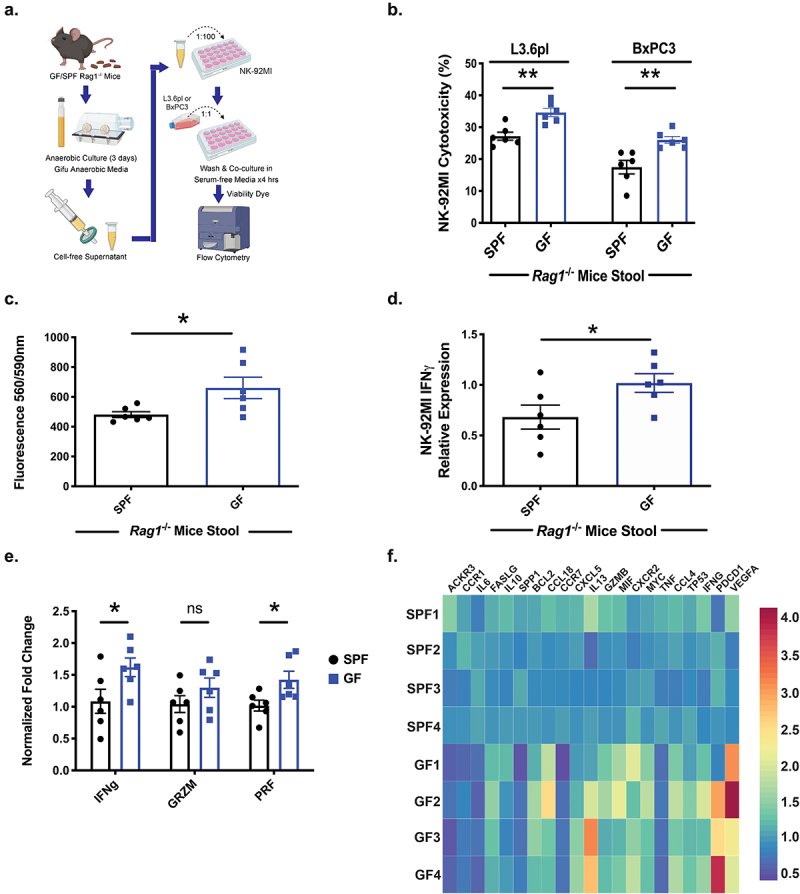
Abiotic bacterial culture supernatant modulates NK cell anti-PDAC activity *in vitro*. Stool was collected from germ-free (GF) and SPF-*Rag1^−/−^* mice and cultured anaerobically for 3 days. Abiotic culture supernatant was prepared after filtration and NK-92MI cells treated with 1% of this supernatant for 3 days. After exposure, NK-92MI cells were harvested and then co-cultured with the human PDAC cell line L3.6pl or BxPC3 for 4 hours and flow cytometry performed to determine cytotoxicity as described (a). NK cells exposed to abiotic culture supernatant from SPF-derived stool had decreased cytotoxicity to L3.6pl and BxPC3 compared to GF-derived abiotic supernatant *in vitro* (b). Furthermore, NK-92MI cells had a decreased migratory ability in a Boyden chamber assay when pre-treated with abiotic culture supernatant from SPF-*Rag1*^−/−^ stool (c). These findings of decreased cytotoxicity and decreased migration correlated with decreased activation of NK-92MI cells as measured by flow cytometry for IFNγ(+) NK-92MI cells after exposure to abiotic culture supernatant from SPF-*Rag1*^−/−^ stool (d). To interrogate pathways responsible for altered cytoxicity, qPCR of treated NK-92MI cells again demonstrated decreased NK cell activation (IFNγ+) but also decreased Perforin expression when exposed to abiotic culture supernatant from SPF stool (e). Furthermore, decreased NK cell cytotoxicity and migration pathways were found via qPCR array as well as increased expression of inhibitory NK cell pathways in NK-92MI cells treated with abiotic culture supernatant from SPF stool (f). (*) *p* < .05, (**) *p* < .01.

While we, and others, have demonstrated intrapancreatic bacteria in human PDAC samples,^8,10^ the importance of this phenomenon is unknown since PDAC xenografts lacking intratumoral bacteria are still responsive to the state of the intestinal microbiota.^10^ We further confirmed, as previously reported,^10^ that xenografts from both heterotopic L3.6pl and orthotopic Pan02 are devoid of detectable bacteria by qPCR analysis (Fig. S7). Therefore, we hypothesized that the gut microbiota modulates NK cell anti-PDAC activity independent of direct microbial interaction within the tumor microenvironment. To test this hypothesis, we prepared abiotic supernatant from anaerobic and aerobic cultured SPF or GF-*Rag1^−/−^* mouse stool. Human NK-92MI cells were pre-treated with abiotic culture supernatant to determine cytotoxicity to L3.6pl and BxPC3 human PDAC cells *in vitro* (Figure 3a). When compared to the abiotic anaerobic culture supernatant from GF-*Rag1*^−/−^ fecal pellets, the NK-92MI cells treated with abiotic anaerobic culture supernatant derived from SPF-*Rag1*^−/−^ stool had a 51% reduced cytotoxicity against L3.6pl PDAC cells (*p* = .087) and 32.8% reduction against BxPC3 cells (*p* = .002, Figure 3b). There was no statistical difference in cytotoxicity between aerobic culture supernatant and GF-derived stool culture supernatant (data not shown). An essential part for NK cell anti-tumor function is the ability to migrate and infiltrate the tumor microenvironment. Thus, we next tested the effect of abiotic culture supernatant on NK cell migration. NK-92MI cells treated with abiotic culture supernatant from SPF-*Rag1^−/−^* stool had 26% decreased migration ability compared to treatment with abiotic supernatant derived from GF-*Rag1^−/−^* stool (*p* = .026, Figure 3c). This reduction in cytotoxicity and migration correlated with decreased NK-92MI cell activation as evidenced by a 33.1% reduction in IFNγ expression upon exposure to abiotic culture supernatant derived from SPF-*Rag1*^−/−^ stool compared to GF-*Rag1*^−/−^ stool (Figure 3d). Subsequently, qPCR analysis for NK cell killing pathways was performed and again demonstrated decreased IFNγ expression (*p* = .048) as well as Perforin (*p* = .015) in NK-92MI cells exposed to abiotic stool culture supernatant derived from SPF-*Rag1^−/−^* compared to GF-*Rag1^−/−^* mice. (Figure 3e). Finally, these findings were further confirmed by a qPCR array, which indicated that NK-92MI cells treated with abiotic culture supernatant from SPF-*Rag1*^−/−^ stool had decreased gene expression of NK cell activation/migration pathways including FASLG, CCL18, IL-13, CXCR2, CCL4, and increased expression associated with inhibition of NK cell activity including CCR1 and IL-6 (figure 3f, Supplemental Table 1). These data suggest a modulatory impact of gut microbiota-derived components on NK cell activation and associated anti-tumor activities.

## Discussion

In this study, we demonstrate that the gut microbiota inhibits intratumoral NK cell infiltration and activation with resultant increased PDAC progression. NK cells are important for gut microbiota-mediated PDAC progression in immunodeficient and immunocompetent murine models given that antibody-mediated NK cell depletion in both models resulted in advanced tumors despite the lack of a microbiota in antibiotic-treated mice.

Our findings that innate immunity is important for PDAC control is supported by numerous lines of evidence. In addition to our previous work with Nod-SCID mice,^10^ herein we utilized a second immunodeficient model, *Rag1^−/−^* mice, combined with two different microbiota conditions: GF and antibiotic-mediated microbiota depletion. While data has shown increased intratumoral Th1 cells in antibiotic-treated mice, which could result in increased IFNγ production,^8^ this potential confounder was addressed both by the use of *Rag1*^−/−^ mice, which lack Th1 cells, and GF mice for which antibiotic microbiota depletion was not necessary. Both approaches demonstrated that innate immunity is important for microbiota-mediated PDAC development. Thus, in addition to the well-established anti-tumor role of the adaptive immune system in PDAC,^32^ our study demonstrates that innate immunity, specifically, the NK cell component, is also an important player in bacteria-mediated tumor immune response. Although *Rag1*^−/−^ mice may raise the potential for altered NK cell response to PDAC tumors, the data generated using immunocompetent C57BL/6 J mice harboring a syngeneic PDAC cell line, in conjunction with depletion of NK cells in both immunocompromised and immunocompetent models, support the premise that bacteria influence PDAC NK cell infiltration and activation, and subsequently pancreatic cancer progression.

Although intratumoral bacteria may impact the tumor immune environment and cancer development,^8,33^ intratumoral bacteria were not detected in our xenograft models. This finding implies a mechanism connecting intestinal bacteria to distant organs, potentially through microbial-derived components.^24,34^ These microbial-derived components could have deleterious or beneficial impact on host anti-tumor immune response. For example, gut microbiome-mediated bile acid metabolism has been shown to decrease CXCL16 expression in liver sinusoidal endothelial cells, which inhibited CXCR6+ hepatic natural killer T (NKT) cells recruitment and activation.^34^ In contrast, microbiome-derived butyrate can increase liver NKT and Th17 cells but decrease Tregs, which subsequently has been shown to improve the anti-cancer immune response to colorectal cancer liver metastasis.^35^ Our abiotic supernatant assays suggest that microbial-derived component(s) impacts NK cell anti-tumor activities including migration and cytotoxicity, which could explain the pro-tumor effect of the gut microbiota. Such an impact of bacterial soluble components on NK cell anti-tumor function has a prescedent.^36^ For example, byproducts of microbial metabolism of soy isoflavones have been shown to decrease cytokine-induced NK cell function.^37^ Additionally, the gut commensals *Bifidobacterium* and *Lactobacillus* can synthesize folate, which has also been found to modulate NK cell cytotoxicity.^38,39^ Further studies would be necessary to identify specific bacteria species and its derived components that could crosstalk with tumor infiltrating NK cells and subsequent PDAC progression.

In summary, our study demonstrates that the gut microbiota mediates PDAC progression through NK cell modulation and that gut microbiota-derived supernatant can modulate anti-tumor NK cell activity. As such, harnessing the gut microbiota to modulate the innate immune system holds promise for the treatment of patients with pancreatic cancer and modulating the microbiome may be one way to facilitate the goal of improving survival in patients with this deadly disease.

## Materials and methods

### Animal husbandry

The University of Florida (UF) Institutional Animal Care and Use Committee approved all animal experiments and national guidelines were followed (Protocol #202008485). Mixed-gender mice aged 4–6 weeks old were used and housed in a single dedicated specific pathogen-free (SPF) room throughout each experiment unless indicated for gnotobiotic experiments. *Rag1^−/−^* mice were purchased from Jackson Laboratory (Bar Harbor, ME) and acclimated in their SPF room for at least 1 week prior to utilization. In-house C57BL/6 J mouse breeding was performed by dedicated UF Animal Care Service personnel. Where indicated, gut microbiota depletion was accomplished by supplementing the animal drinking water with an antibiotic cocktail (Abx) consisting of 0.5 mg/L metronidazole, 1 gm/L neomycin, 0.5 gm/L vancomycin, and 0.125 gm/L ciprofloxacin. Microbiota depletion was confirmed by routine culture of murine stool and/or 16S quantitative PCR (qPCR) of fecal bacterial DNA. For gnotobiotic experiments, germ-free *Rag1^−/−^* mice (generous gift from Dr. Jeremiah Faith, Mount Sinai) were bred and maintained in gnotobiotic isolators by UF Animal Care Service personnel until ready for use at which time they were transferred into the Techniplast ISOcage P Bioexclusion system (Techniplast; West Chester, PA) and cages were sterilely changed every two weeks per manufacturer protocol.^40,41^

### Bacterial manipulation

To assess the ability of specific microbes to modulate PDAC progression, bacteria were orally gavaged into GF-*Rag1^−/−^* mice at 1*10^8^ colony forming units (CFU) at the time of transfer into the ISOcage system. After two weeks colonization, as confirmed by culture and PCR, 1*10^6^ L3.6pl cells were subcutaneously injected as described. To maintain IsoCage experimental gnotobiotic status, xenografts were only measured at the study endpoint when the xenografts were harvested. The murine bacterial status (GF or microbiota-associated) was confirmed by routine bacterial culture of stool and qPCR of bacterial DNA isolated from stool at the time of each cage change. For fecal transplant experiments, fecal pellets (1 gm) from SPF-*Rag1^−/−^* mice were collected and resuspended in 5 mL sterile PBS. Subsequently, 200 μL of this fecal slurry was orally gavaged into GF mice to create the “Ex-GF” cohort. This cohort was then transferred to and remained in SPF housing for the duration of the experiment.

### NK cell depletion

NK cell depletion in mice was achieved by intraperitoneal injection of 50ug anti-Asialo GM1 antibody (“anti-ASGM1”; Invitrogen-ThermoFisher Scientific; Waltham, MA) twice weekly starting one week before tumor implantation and continued until the experimental endpoint.^42,43^ Controls were treated with an equivalent amount of rabbit polyclonal anti-IgG isotype control antibody. NK cell depletion was confirmed by flow cytometry of single-cell isolations from mouse spleen.

### Statistical analysis

Statistical comparison between two groups was performed using a two-tailed Mann–Whitney U-test while multi-group comparisons were performed by ANOVA. *p* ≤ .05 was considered statistically significant. Data were graphed and analyzed using GraphPad Prism 6 (GraphPad Software; San Diego, CA). Graphs depict mean data ±SEM, unless indicated otherwise.

## Supplementary Material

Supplemental Material

## Data Availability

The authors confirm that all data supporting the findings of this study are available within the article and its supplementary materials. Raw data that support the findings of this study are available from the corresponding author [RMT], upon reasonable request.
