# Supplementary material for: Intestinal microbiota modulates pancreatic carcinogenesis through intratumoral natural killer cells
Source: Gut Microbes. 2022 Aug 18;14(1):2112881. doi: 10.1080/19490976.2022.2112881 (PMC9397420; doi:10.1080/19490976.2022.2112881)
Supplement: Supplemental Material [file KGMI_A_2112881_SM2468.zip › NK Paper Gut Microbes Supplemental materials RESUBMISSION_MarkedChangesFINAL.docx]

**Supplemental Materials**

**Materials and Methods**

*Cell Culture and Maintenance*

The human pancreatic cancer cell line, L3.6pl, and the murine pancreatic cancer cell line, Pan02 (National Cancer Institute Division of Cancer Treatment & Diagnosis; Frederick, MD), were used for murine xenograft induction.^1,2^ The human pancreatic cancer cell line, BxPC3, was additionally used for *in vitro* experiments. Cell lines were authenticated by ATCC through their STR profiling cell authentication service and confirmed negative for mycoplasma by PCR. Cancer cell lines were cultured in Dulbecco’s Modified Eagle Medium (L3.6pl, BxPC3) or RPMI (Pan02) with 10% fetal bovine serum and 1% penicillin/streptomycin. The human NK cell line, NK-92MI, was cultured in Alpha Minimum Essential Medium (Sigma-Aldrich; St. Louis, MO) supplemented with 2mM L-glutamine, 1.5gm/L sodium bicarbonate, 0.2mM inositol, 0.1mM 2-mercaptoenthanol, NK-92MI cells have been used in cancer-related investigations for their reproducibility, ease of culture, and lack of sorting/purification needs.^3–5^ 0.02mM folic acid, 1% penicillin/streptomycin, 12.5% horse serum, and 12.5% fetal bovine serum. All cell lines were maintained in a humidified incubator at 37°C with 5% CO_2_.

*Xenograft Models*

Xenografts were generated either heterotopically (1*10^6^ L3.6pl subcutaneous flank inoculation into *Rag1^-/-^* mice) or orthotopically (1*10^6^ Pan02 inoculated intrapancreatic in syngeneic C57BL/6J mice) using fresh, sterile instruments for each mouse in a laminar flow hood after procedure site sterilization with 4% (w/v) chlorhexidine gluconate solution (Mölnlycke Health Care; Gothenburg, Sweden). Heterotopic xenograft growth was assessed twice weekly with a digital caliper to measure the maximum length (L) and width (W) in situ, and the volume estimated by [V=(L^2^)(W/2)]. At study endpoint, xenografts were sterilely harvested in a laminar flow hood and xenografts measured in 3 dimensions to calculate the final volume. Volumes for orthotopic Pan02 xenografts were calculated at the study endpoint after sterile harvesting as described. All fecal and tissue specimens were sterilely harvested in a laminar flow hood and stored in sterile tubes at -80°C until use.

*Stool/Bacterial Culture and Abiotic Supernatant Extraction*

Stool samples from germ-free (GF) or specific pathogen-free (SPF) *Rag1^-/-^* mice were collected into sterile containers in a laminar flow hood and cultured immediately in non-selective Gifu Anaerobic Broth (HiMedia; Chester, PA) in a type B vinyl anaerobic chamber (Coy Laboratory; Grass Lake, MI) for 72 hours or in an aerobic chamber for 24 hours. For studies involving the use of culture supernatants, the individual stool cultured supernatants were then transferred and filtered through 0.22um syringe filters (Olympus Life Science; Waltham, MA) to remove live/dead bacteria and debris to create the abiotic supernatant. Abiotic supernatants were used immediately for subsequent NK cell stimulation studies.

*NK Cell Cytotoxicity*

To test the ability of bacteria culture supernatant to modulate NK cell cytotoxicity to PDAC cells *in vitro*, 5*10^4^ NK-92MI cells were seeded into 24-well plates and 1% (v/v) stool-derived abiotic supernatants were added to the culture media. After 3 days of exposure to the supernatant, NK-92MI cells were collected, counted, and resuspended in fresh serum-free culture media. Co-culture of these supernatant-exposed NK-92MI cells with equal amount of GFP-expressing L3.6pl cells was performed for 4 hours in serum-free NK cell culture media. Floating cells were collected, and the adherent cells were trypsinized and harvested as well. The entire collection of cells was washed in sterile PBS and the cell mixture was suspended in 100μL PBS and stained with LIVE/DEAD Fixable Violet dye (ThermoFisher Scientific; Waltham, MA) for 15 minutes on ice following 2 wash steps with PBS. The stained cells were immediately evaluated by flow cytometry. LIVE/DEAD Fixable Violet dye negative and GFP-positive cells were gated as the percentage of live L3.6pl cells or the tumor cells that survived NK cell killing. The cytotoxicity, calculated as the percent of target cell death, was determined by subtracting the gated percentage of live L3.6pl cells from the baseline control. Flow cytometry was performed on a BD LSRFortessa flow cytometer (BD Biosciences; San Diego, CA) and analyzed using FlowJo software version 10.5.1 (FlowJo, LLC; Ashland, OR). This flow cytometry based NK cell cytotoxicity assay was modified based on the method of Kandarian et al.^6^

*NK Cell Migration*

To test the ability of abiotic stool culture supernatant to modulate NK cell migratory ability, cell migration was measured with the use of a Transwell migration chamber (pore size, 8 μm; Falcon). A total of 5*10^4^ NK-92MI cells in 200μl of NK cell serum-free medium were seeded in the top of a Transwell chamber while 400μl of serum-free medium was added to the bottom of each well in a 24-well plate. Abiotic anaerobic-cultured stool supernatants were added to the bottom well with 1/100 dilution. After 24 hours incubation at 37°C under 5% CO2, the top inserts were removed and 10% alamarBlue (ThermoFisher) was added to each bottom well to stain migrated NK-92MI cells that were in suspension. After incubation for 4 hours at 37°C under 5% CO2, fluorescence was measured with a microplate fluorescence reader using an excitation wavelength 560nm and an emission wavelength of 590nm.

*Bacterial DNA Extraction and Quantitative PCR Analysis*

Stool samples were suspended in 750μL lysis buffer (200mM NaCl, 100mM Tris pH=8, 20mM EDTA) containing 20mg/mL lysozyme and incubated at 37°C for 30 minutes. The cells were then further lysed by adding 85μL 10% SDS and 20μL proteinase K and incubated at 60°C for 30 minutes. Lysed samples were mixed with phenol (ThermoFisher Scientific; Waltham, MA) and disrupted with 0.1mL of 0.1mm zirconium beads using a Precellys 24 (Bertin Instruments; Montigny-le-Bretonneux, France). The mixtures were centrifugated at 10,000g for 5min and supernatants were then transferred to a new sterile tube for DNA precipitation. The DNA was separated by adding 24:1 chloroform:isoamyl alcohol (Sigma) followed by centrifugation at 14,000g for 5 minutes and then precipitated by the addition of 0.1x volume of 3M sodium acetate and 2x volume of ethanol. The precipitated DNA was then resuspended in 200ul of 10mM Tris buffer (pH 8.0) and the quality further optimized with the Qiagen DNeasy Blood and Tissue Kit (Qiagen; Hilden, Germany). DNA concentration and quality were determined using a NanoDrop 2000 spectrophotometer (ThermoFisher Scientific; Waltham, MA).

Bacterial colonization was determined by quantitative PCR (qPCR) using 16S primers. Briefly, 10μL reaction mixture containing 10ng sample DNA, 100nmol/L paired primers and 2x Power SYBR Green master mix (Applied Biosystems; Foster City, CA) was loaded on the CFX384 Real-Time PCR Detection System (Bio-Rad Laboratories; Hercules, CA) in triplicate. The V1-V3 hypervariable region of the 16S rRNA gene was amplified by using primers 27F (5’-AGAGTTTGATCCTGGCTCAG-3’) and 534R (5′-ATTACCGCGGCTGCTGG-3′) as described.^7^ The bacterial gene expression was determined by qPCR following the program: initial denaturation at 94°C for 10 minutes, followed by 40 cycles at 94°C for 1 minute, annealing at 57°C for 45 seconds, and elongation at 72°C for 1 minute, followed by a final elongation at 72 °C for 5 minutes.

*Flow Cytometry*

For creation of single-cell suspensions from harvested tissue (heterotopic or orthotopic xenografts, spleen) to be used in flow cytometry experiments, each tissue sample was finely minced with a sterile scalpel in a laminar flow hood and kept in cold PBS buffer containing 2mg/ml Stemxymel (Worthington Biochemical Corp.; Lakewood, NJ) and 0.1mg/ml DNase I (StemCell Technologies; Cambridge, MA). Tissues were then incubated at 37°C for 15 minutes and transferred to 25C tubes (Miltenyi Biotec; Bergisch Gladbach, Germany) for homogenization by the gentleMax dissociator (Miltenyi Biotec). Dissociated tissue was then passed through a 100μm cell strainer by centrifugation at 300g for 5 minutes. The cell pellet was washed twice and resuspended in cold cell staining buffer (Biolegend; San Diego, CA). The dissociated cells were preincubated with purified anti-CD16/CD32 mAb (Biolegend; San Diego, CA) to block FcγRIII/II receptor for 15 minutes at 4°C. To exclude the dead cells from analysis, LIVE/DEAD Fixable Violet stain (ThermoFisher Scientific) was added to the cell pellets and incubated for 15 minutes in the dark at 4°C. Cells were then labeled with the indicated cellular surface antibodies for 15 minutes at 4°C. The following antibodies were used for flow cytometry analysis: anti-CD45 (30-F11, Biolegend; San Diego, CA), anti-CD3 (17A2, Biolegend), anti-NK1.1 (PK136, Biolegend), anti-CXCR2 (SA044G4, Biolegend), anti-NKp46 (29A1.4, Biolegend), anti-NKG2D (CX5, Biolegend), anti-CCR7 (4B12, Biolegend), anti-CD107a (1D4B, Biolegend). For quantification of NK cell produced IFNγ, the dissociated single cell suspensions were incubated with 1x Brefeldin A solution (Biolegend) and 1x cell stimulation cocktail (ThermoFisher Scientific) for 4 hours prior to the staining. Intracellular staining was performed using the fixation/permeabilization kit (BD Biosciences). Flow cytometry was performed on a BD LSRFortessa flow cytometer (BD Biosciences) and analyzed using FlowJo software version 10.5.1 (FlowJo, LLC; Ashland, OR). A total of 10^4^ evaluable events (NK cells) were quantified for each flow cytometry run. For some xenografts, 10^4^ events were unable to be analyzed because of small xenografts not containing enough dissociated cells for analysis and is responsible for some differences in reported cohort sizes.

*Transcriptomic Profiling*

RNA was extracted from NK-92MI cells by using the RNeasy Mini Kit (Qiagen; Hilden, Germany) following the manufacturer’s protocol. RNA concentration and quality were determined using a NanoDrop 2000 spectrophotometer. A qPCR array was performed using the RT2 Profiler “Cancer Inflammation and Immunity Crosstalk” PCR Array (Qiagen; Hilden, Germany) following manufacturer’s protocol. Gene expression for human IFNγ expression was also determined by qPCR following the program: initial denaturation at 94°C for 10 minutes, 40 cycles at 94°C for 1 minute, annealing at 57°C for 45 seconds, elongation at 72°C for 1 minute, and followed by a final elongation at 72 °C for 5 minutes. Primers used in the study included: 18S rRNA [HU-18S-F (5’-TGCATGGCCGTTCTTAGTTG-3’), HU-18S-R (5’-AGTTAGCATGCCAGAGTCTCGTT-3’)], human IFNγ [HU-IFNG-F (5’-TGACCAGAGCATCCAAAAGA-3’), HU-IFNG-R (5’-CTCTTCGACCTCGAAACAGC-3’)], mouse beta-actin [M-βActin-F (5’-AGAGGGAAATCGTGCGTGAC-3’), M-βActin-R (5’-CAATAGTGATGACCTGGCCGT-3’)]. Relative gene expression was calculated by using the -2^ΔΔCt^ methodology.^8^

**Supplemental Figure Legends**

**Figure S1. Transcriptomic profile predicts decreased intratumoral NK cell infiltration which associates with decreased overall survival in pancreatic cancer patients.**

RNA sequencing data from L3.6pl PDAC xenografts grown in SPF-Nod-SCID mice or Nod-SCID mice whose microbiota was depleted with antibiotics^9^ was analyzed with the TIMER 2.0 immune estimation algorithm, CIBERSORT.^10,11^ This predicted decreased NK cell infiltration in control (microbiota intact) mice PDAC tumors compared to antibiotic treated **(A)**. Decreased NK cell infiltration also associates with decreased overall survival in pancreatic cancer patients on Kaplan-Meier logistic regression analysis based on data gathered from The Cancer Genome Atlas database **(B).**

**Figure S2. Confirmation of intestinal microbiota status by 16S DNA PCR.**

The intestinal microbiota status of *Rag1*^-/-^ mice was confirmed in representative samples by 16S rDNA PCR and demonstrated efficient depletion of the intestinal microbiota in mice treated with an antibiotic cocktail (Abx) versus specific pathogen-free (SPF) mice **(A)** and absence of bacteria in germ-free (GF) mice compared to SPF and GF mice gavaged with stool derived from SPF-*Rag1*^-/-^ mice **(B)**.

**Figure S3**. **Presence of the gut microbiota associates with decreased PDAC tumor growth in immunocompromised and immunocompetent mice.**

The L3.6pl PDAC xenografts in *Rag1^-^*^/-^ mice exhibited increased volume and weight in mice with an intact microbiota in both SPF vs. Abx **(A)** and SPF/Ex-GF vs. GF **(B)**. Immunocompetent C57BL/6J mice likewise had increased Pan02 tumor volume and weight in microbiota-intact SPF mice compared to those in which their microbiota was depleted with Abx **(C)**. (*) *p* < 0.05, (**) *p* < 0.01.

**Figure S4. Microbiota-intact C57BL/6J mice bearing orthotopic PDAC xenografts had a trend toward decreased CD4+ and CD8+ T-cell infiltration compared to microbiota-depleted.**

Flow cytometry of dissociated Pan02 xenografts from C57BL/6J mice demonstrated decreased, but not statistically significant, CD4+ **(A)** and CD8+ **(B)** T-lymphocytes in tumors grown in mice with intact microbiota (SPF) mice compared to antibiotic (Abx)-mediated microbiota depleted mice. (ns) not significant.

**Figure S5. Flow cytometry gating strategy for immune profiling.**

Flow cytometry gating strategy used for immune profiling of PDAC xenografts (L3.6pl and Pan02) with representative figures from Pan02 orthotopic pancreatic xenograft in SPF-C57BL/6J mice illustrated.

**Figure S6. Treatment of *Rag1*^-/-^ with anti-ASGM1 antibody effectively eliminated NK cells in the spleen and implanted L3.6pl PDAC xenografts.**

The ability of intraperitoneal (IP) administered anti-Asialo GM1 (antio-ASGM1) to deplete the NK cell population in spleen **(A)** and L3.6pl PDAC xenografts **(B)** in *Rag1^-/-^* mice was confirmed by flow cytometry versus anti-IgG isotype control antibody.

**Figure S7.**

Heterotopic L3.6pl PDAC xenografts in *Rag1*^-/-^ mice **(A)** and orthotopic Pan02 syngeneic PDAC xenografts in C57BL/6J mice **(B)** did not harbor intratumoral bacteria in either the control (SPF, microbiota-intact) or Abx-mediated microbiota-depleted mice in a representative cohort of samples.

**Supplemental Table 1. Natural killer cell gene expression values from the quantitative PCR array.**

**Supplemental References**

1. Bruns CJ, Harbison MT, Kuniyasu H, et al. In vivo selection and characterization of metastatic variants from human pancreatic adenocarcinoma by using orthotopic implantation in nude mice. Neoplasia 1999;1:50–62.

2. Pushalkar S, Hundeyin M, Daley D, et al. The pancreatic cancer microbiome promotes oncogenesis by induction of innate and adaptive immune suppression. Cancer Discov. 2018;8:403–416.

3. Francescone R, Barbosa Vendramini-Costa D, Franco-Barraza J, et al. Netrin G1 Promotes Pancreatic Tumorigenesis through Cancer-Associated Fibroblast-Driven Nutritional Support and Immunosuppression. Cancer Discov. 2021;11:446–479.

4. Tseng H-C, Xiong W, Badeti S, et al. Efficacy of anti-CD147 chimeric antigen receptors targeting hepatocellular carcinoma. Nat. Commun. 2020;11:4810.

5. Judge SJ, Dunai C, Aguilar EG, et al. Minimal PD-1 expression in mouse and human NK cells under diverse conditions. J. Clin. Invest. 2020;130:3051–3068.

6. Kandarian F, Sunga GM, Arango-Saenz D, et al. A Flow Cytometry-Based Cytotoxicity Assay for the Assessment of Human NK Cell Activity. J. Vis. Exp. 2017.

7. Tomkovich S, Dejea CM, Winglee K, et al. Human colon mucosal biofilms from healthy or colon cancer hosts are carcinogenic. J. Clin. Invest. 2019.

8. Livak KJ, Schmittgen TD. Analysis of relative gene expression data using real-time quantitative PCR and the 2(-Delta Delta C(T)) Method. Methods 2001;25:402–408.

9. Thomas RM, Gharaibeh RZ, Gauthier J, et al. Intestinal microbiota enhances pancreatic carcinogenesis in preclinical models. Carcinogenesis 2018;39:1068–1078.

10. Li T, Fan J, Wang B, et al. TIMER: A Web Server for Comprehensive Analysis of Tumor-Infiltrating Immune Cells. Cancer Res. 2017;77:e108–e110.

11. Li T, Fu J, Zeng Z, et al. TIMER2.0 for analysis of tumor-infiltrating immune cells. Nucleic Acids Res. 2020;48:W509–W514.
