# Supplementary figures and images for: Intestinal microbiota modulates pancreatic carcinogenesis through intratumoral natural killer cells
Source: Gut Microbes. 2022 Aug 18;14(1):2112881. doi: 10.1080/19490976.2022.2112881 (PMC9397420; doi:10.1080/19490976.2022.2112881)

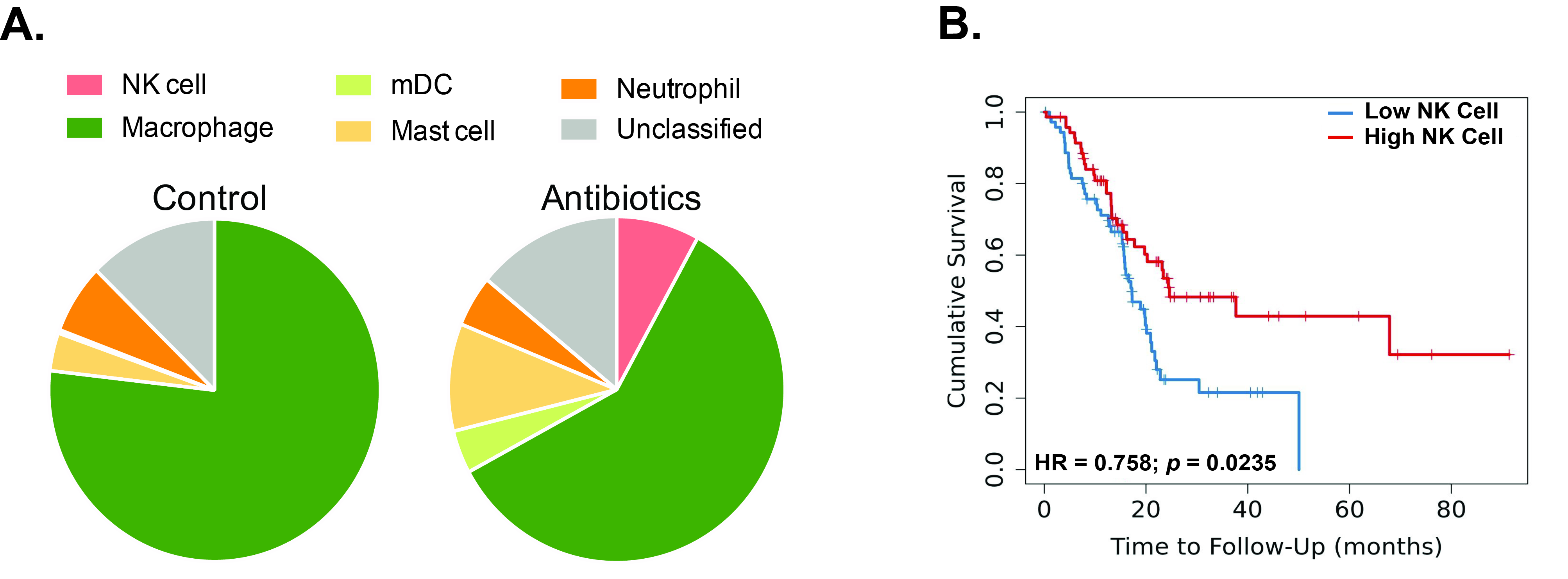

Supplement: Supplemental Material [file KGMI_A_2112881_SM2468.zip › Gut Microbes RESUBMISSION Supp Fig 1.tif]

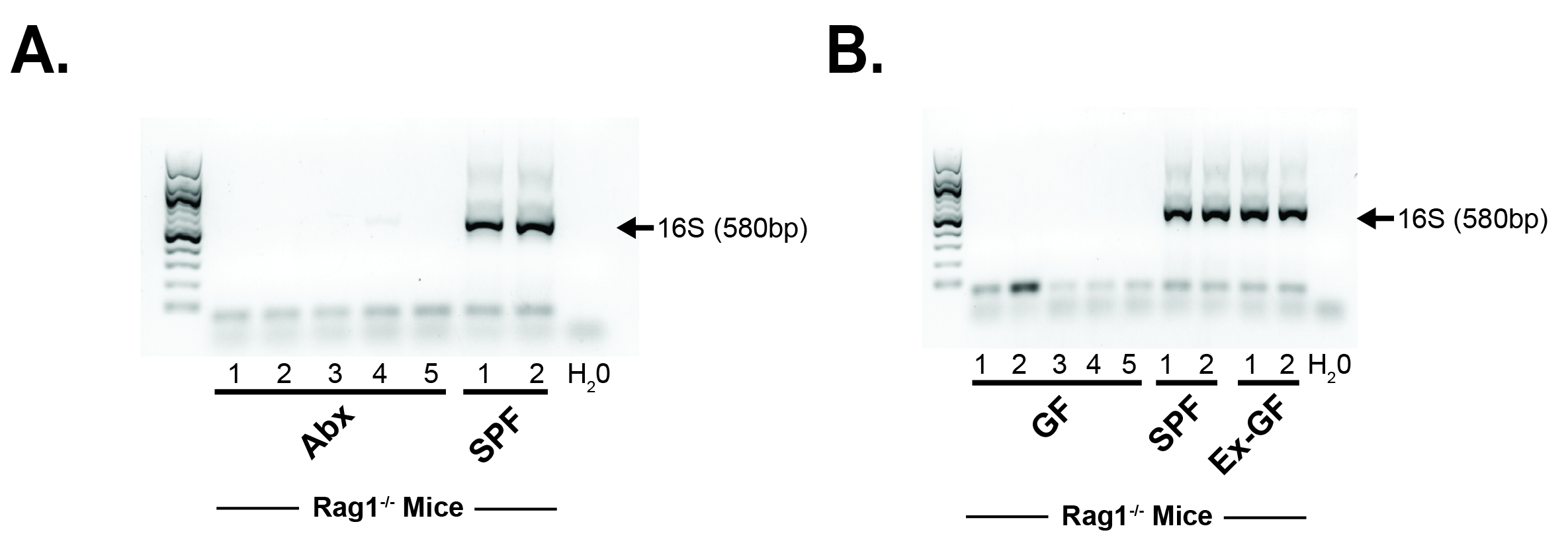

Supplement: Supplemental Material [file KGMI_A_2112881_SM2468.zip › Gut Microbes Supp Fig 2.tif]

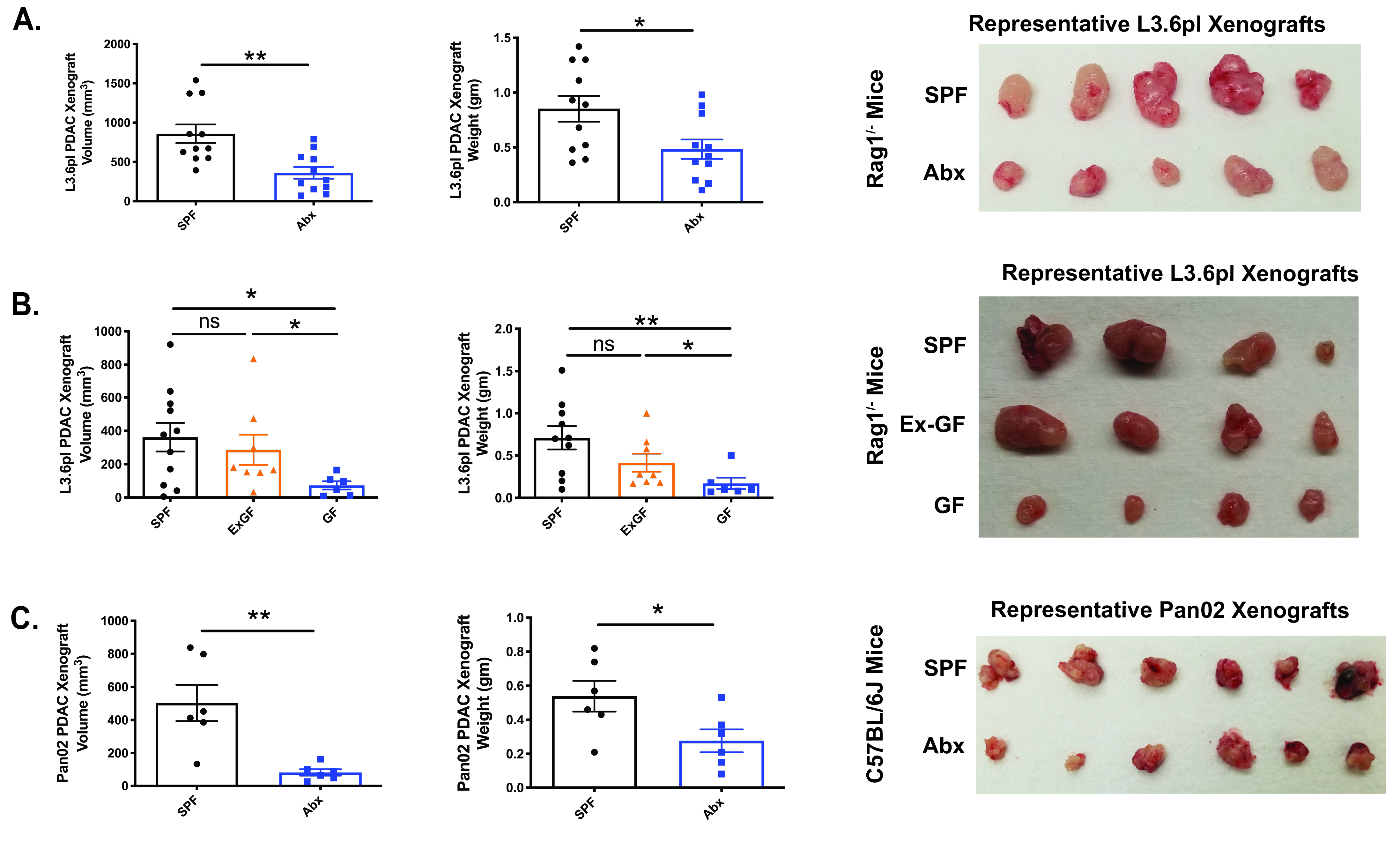

Supplement: Supplemental Material [file KGMI_A_2112881_SM2468.zip › Gut Microbes Supp Fig 3.tif]

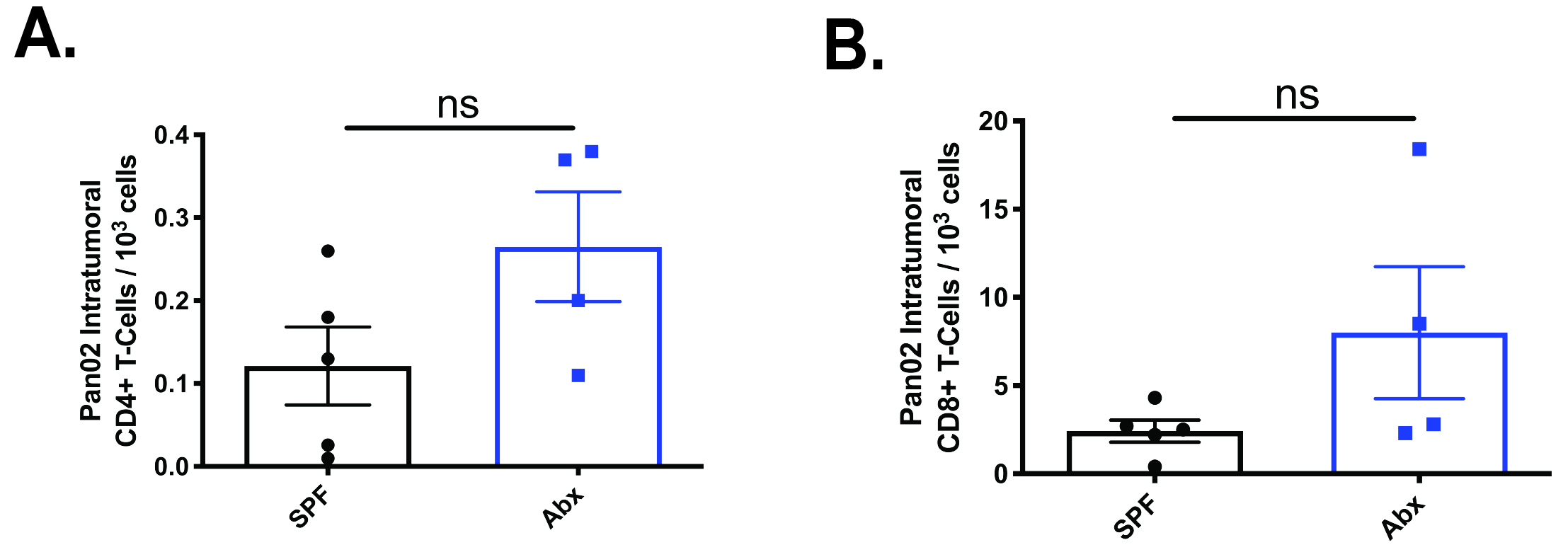

Supplement: Supplemental Material [file KGMI_A_2112881_SM2468.zip › Gut Microbes Supp Fig 4.tif]

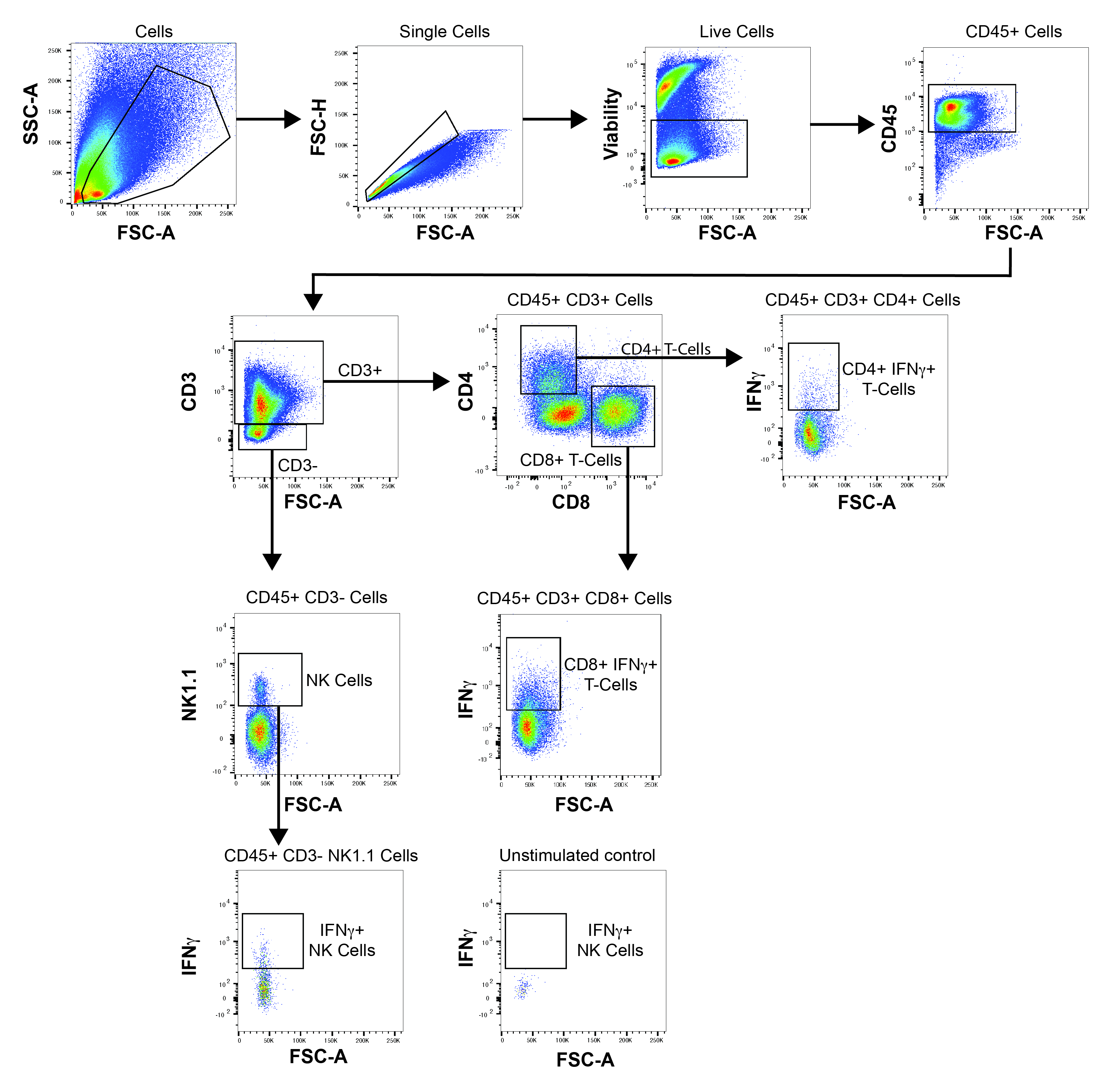

Supplement: Supplemental Material [file KGMI_A_2112881_SM2468.zip › Gut Microbes Supp Fig 5.tif]

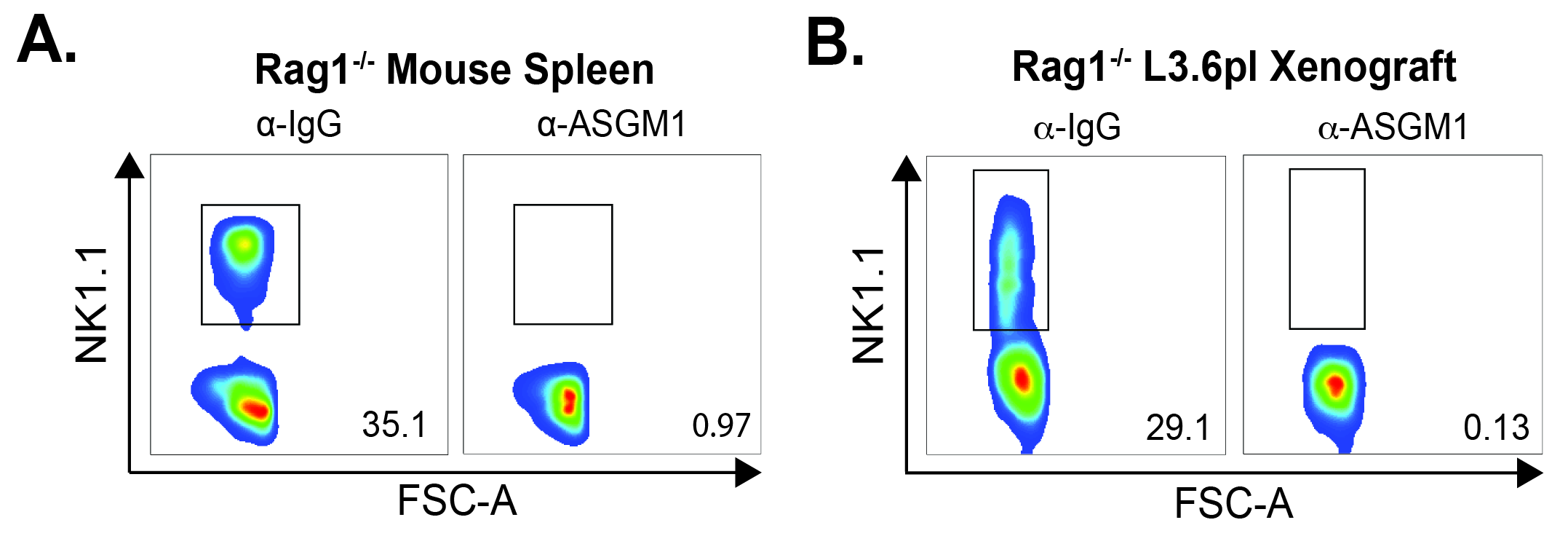

Supplement: Supplemental Material [file KGMI_A_2112881_SM2468.zip › Gut Microbes Supp Fig 6.tif]

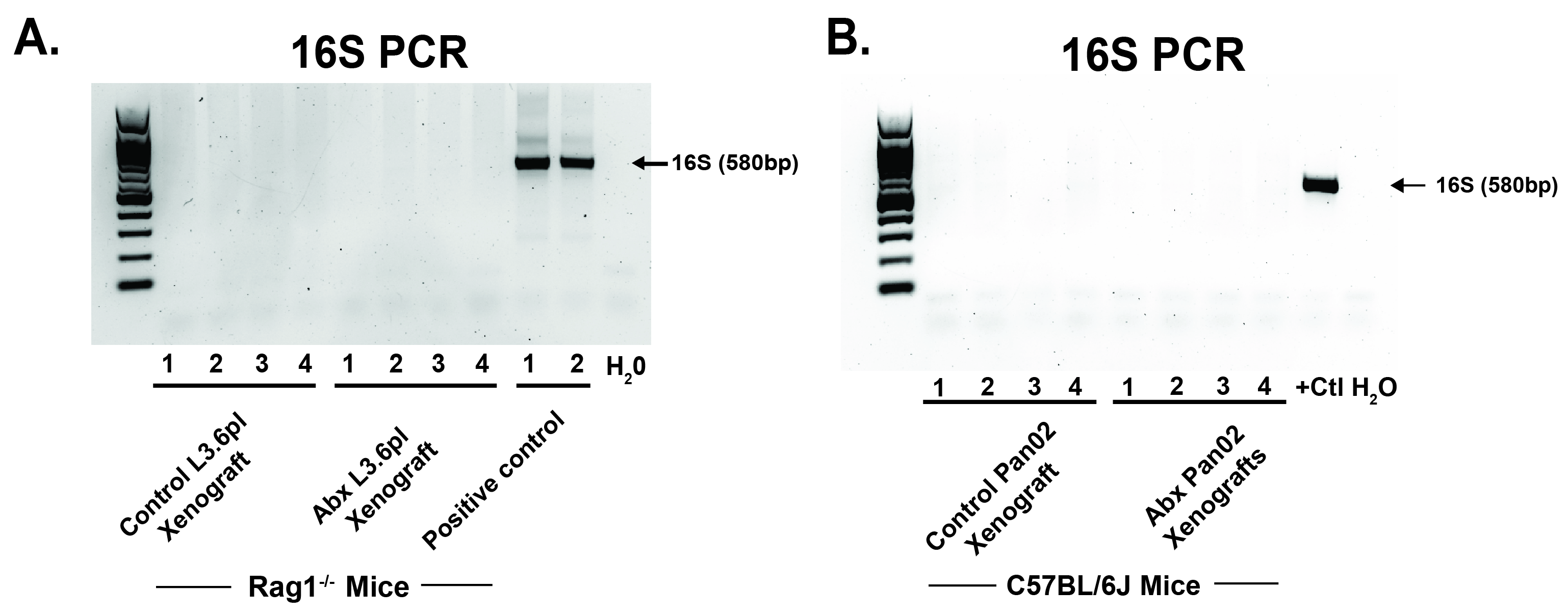

Supplement: Supplemental Material [file KGMI_A_2112881_SM2468.zip › Gut Microbes Supp Fig 7.tif]
